# Detection of Cytomegalovirus in Patients Attending a Tertiary Care Center: A Retrospective Analysis

**DOI:** 10.7759/cureus.104351

**Published:** 2026-02-27

**Authors:** Sivacchandran Subbarayan Rajasekaran, Udhaya Devi Dinakaran, Shanthi Mariappan, Ramya Barani

**Affiliations:** 1 Microbiology, Sri Ramachandra Institute of Higher Education and Research, Chennai, IND

**Keywords:** cmv-dna, cytomegalovirus, immunosuppression, neonates, transplant recipient, viral load

## Abstract

Introduction

Human cytomegalovirus (CMV) is a DNA β-herpesvirus that establishes lifelong latency in bone marrow-derived CD34+ progenitors and CD14+ monocytes following primary infection. The virus restricts gene expression to prevent replication but reactivates when these cells differentiate into macrophages or dendritic cells, often triggered by inflammation or immunosuppression, and causes significant morbidity. Congenital CMV remains a major global health issue and the leading cause of non-genetic neurodevelopmental impairment worldwide. Compared to seroprevalence studies, fewer studies have evaluated active CMV infection using molecular methods. Quantitative real-time polymerase chain reaction (qPCR)-based detection of CMV DNA provides a reliable measurement of active infection and viral burden.

Objectives

This study was conducted to assess the prevalence, demographic characteristics, and viral load distribution of CMV and to evaluate its clinical spectrum and association with underlying diseases in patients with suspected infection.

Materials and methods

This retrospective analysis was conducted at a tertiary care hospital in Chennai, India, involving 162 patients who were tested from January 2024 to June 2024. Viral DNA was extracted using spin-column extraction kits (Qiagen Blood Mini-kits). CMV DNA detection and quantification were performed using a TaqMan-based real-time PCR assay (Thermo Fisher Scientific, Waltham, MA, USA), targeting the CMV ppUL83 gene on a Rotor-Gene-Q instrument (Qiagen, Hilden, Germany). The lower limit of detection was 50 copies/mL.

Results

In our study, CMV DNA was detected in 43 (26.5%) of the 162 patients. Among the CMV-positive patients, age and gender showed no significant association. Preterm birth was noted in 12 (27.9%) patients. Immunocompromised individuals showed a significant association with CMV positivity; among these, seven (16.3%) patients were transplant recipients, and five (11.6%) patients had hematological malignancies. Gastrointestinal manifestations were the most common presenting feature, seen in 22 (51.1%) patients. Of the 43 CMV-positive individuals, 23 (53.5%) had viral loads ranging from 50 to 10,000 viral DNA copies/mL.

Conclusion

CMV infection represents a significant health burden in tertiary care settings. This study highlights the role of qPCR in the early detection of active CMV infections. Early diagnosis allows timely initiation of antiviral therapy and thereby improves clinical outcomes, particularly in immunocompromised individuals.

## Introduction

Human cytomegalovirus (CMV) is an enveloped, double-stranded DNA virus of the family Herpesviridae (subfamily *Betaherpesvirinae*). In immunocompetent individuals, primary CMV infection is often asymptomatic or presents as a mild, self-limited influenza-like illness or mononucleosis-like syndrome [1]. Following primary infection, CMV establishes lifelong latency with periodic reactivation, particularly when cell-mediated immunity is impaired. Both primary infection and reactivation can lead to severe, organ-specific disease and significant morbidity in immunocompromised hosts [2].

CMV remains a major opportunistic pathogen in solid organ and hematopoietic stem cell transplant recipients, patients with malignancies, and individuals receiving immunosuppressive therapies [3]. In these patients, CMV can manifest as pneumonitis, hepatitis, colitis, retinitis, or disseminated disease. It also contributes to indirect effects such as heightened susceptibility to other infections and adverse graft outcomes [4]. Among kidney transplant recipients, CMV infection typically occurs early after transplantation in the absence of prophylaxis, and delayed-onset disease can occur even after antiviral prophylaxis. The incidence and severity of CMV disease are influenced by the type and intensity of immunosuppression, CMV serostatus of donor and recipient, and the transplanted organ type [5]. CMV infection has been associated with acute rejection and chronic allograft dysfunction. This highlights the importance of timely diagnosis and appropriate management in transplant settings [6].

Apart from transplant-related disease, CMV is the most common congenital viral infection worldwide, with an estimated incidence of approximately 0.6%-0.7% of live births. It also remains an important cause of sensorineural hearing loss and neurodevelopmental delay [7,8]. Early diagnosis during the newborn period is essential, as timely recognition and treatment in selected cases may reduce disease burden and improve outcomes [8,9].

Globally, CMV seroprevalence exceeds 80%, with higher rates reported from developing countries, including India [10]. In high-seroprevalence settings, serological assays have limited use for diagnosing active disease because they cannot accurately distinguish latent infection from active viral replication, and interpretation may be affected by baseline immunity. Therefore, detection and quantification of CMV DNA using real-time quantitative polymerase chain reaction (qPCR) have become central to clinical practice [11]. qPCR allows sensitive identification of CMV DNAemia and provides viral load quantification for risk assessment. It also helps in decision-making regarding prophylactic or pre-emptive therapy, and allows objective monitoring of treatment response [12].

It has been reported that the gastrointestinal manifestations of CMV mimic inflammatory bowel disease (IBD) [13]. CMV infection occurring in transplant recipients despite prophylaxis has also been reported [6]. In resource-limited settings, diagnostic access and systematic viral load-based screening may be variable, and may lead to delayed recognition and underestimation of the disease burden.

Against this background, the present study was undertaken to detect active CMV infection using real-time PCR, quantify CMV viral load, and assess its prevalence, demographic characteristics, clinical spectrum, and underlying conditions of the affected patients. This will contribute to strengthening diagnostic and management strategies in the study setting.

## Materials and methods

Study design and setting

We performed a retrospective study at the Molecular Biology and Clinical Virology Division of Sri Ramachandra Medical College and Research Institute in Chennai, India. The study covered patient data collected over a period of six months, from January 2024 to June 2024. The study was approved by the Institutional Ethics Committee of Sri Ramachandra Institute of Higher Education and Research (CSP-MED/25/AUG/120/236).

Inclusion and exclusion criteria

The study population included 162 patients with clinical suspicion of CMV infection. The inclusion criteria consisted of neonates with features suggestive of congenital infection and sepsis; immunocompromised adults, including transplant recipients and patients with hematological malignancies; and patients presenting with fever for 7-10 days, gastrointestinal symptoms, or respiratory illness suggestive of viral etiology. Clinical specimens included whole blood (ethylenediaminetetraacetic acid (EDTA)), urine, saliva, and bronchoalveolar lavage samples, depending on clinical indication.

To ensure statistical independence, repeated samples collected from the same patient were excluded.

DNA extraction

DNA was extracted using the QIAamp DNA Mini Kit (Qiagen, Hilden, Germany), following the manufacturer’s instructions. Extracted DNA was used for real-time PCR or stored at -20°C until further use.

Real-time quantitation of CMV

CMV DNA detection and quantification were performed using real-time PCR (TaqMan-based assay; Thermo Fisher Scientific, Waltham, MA, USA), targeting the CMV ppUL83 gene with a commercial CMV kit (R-gene^TM^; bioMérieux, Marcy-l'Étoile, France), using a Rotor-Gene-Q real-time PCR instrument (Qiagen, Hilden, Germany).

According to the manufacturer’s protocol, real-time PCR was carried out in a 0.2 mL PCR tube with a 25 µL reaction volume containing 10 µL of DNA template and 15 µL of CMV PCR amplification premix, in which the target region, the ppUL83 gene, was amplified with a product of 283 base pairs. The lower limit of detection was 50 copies/mL. Samples showing a sigmoid curve amplification with a crossing threshold (Ct) value of 15-35 were considered positive, and the amount of DNA was estimated from a range of standard curves generated during amplification. DNA integrity of the PCR-negative samples was confirmed by the presence of internal control DNA. The test sample results were reported as numeric concentration in copies per milliliter of the sample. Each run included internal controls, positive controls, and negative controls to ensure assay validity.

Statistical analysis

The categorical variables were summarized as numbers (n) and percentages (%). We used Pearson’s chi-square test or Fisher’s exact test, as appropriate, to assess the associations between categorical variables such as gender, clinical presentations, and CMV positivity. For inferential analyses, results are reported as the test statistic (χ^2^) and p-value. A p-value of <0.05 was considered statistically significant. Statistical analysis was conducted using SPSS software version 17 (Released 2018; SPSS Inc., Chicago, IL, USA).

## Results

Association between gender and CMV positivity

Of the 162 patients who were tested, CMV DNA was detected in 43 patients (26.5%). Males accounted for 29% (29/100), while females accounted for 22.6% (14/62). Even though the positivity rate was slightly higher in males, there was no statistically significant association between gender and CMV positivity (χ² = 0.513, p-value = 0.47) (Table 1).

**Table 1:** Comparison of gender distribution between CMV-positive and CMV-negative patients Data are presented as numbers (n) and percentages (%). Association was assessed using Pearson’s chi-square test. A p-value of < 0.05 was considered statistically significant. CMV, cytomegalovirus

Gender	CMV Positive (n = 43)	CMV Negative (n = 119)	χ²-value	p-value
Male	29 (29%)	71 (71%)	0.513	0.474
Female	14 (22.6%)	48 (77.4%)		
Total	43	119		

Age-wise distribution of CMV positivity

Among the 43 positive cases, pediatric patients (1 month-18 years) accounted for 50% (3/6). This was followed by adults (>18 years), accounting for 34.2% (26/76), whereas the late neonates (7-28 days) and early neonates (0-6 days) accounted for 18.2% (2/11) and 17.4% (12/69), respectively (Table 2).

**Table 2:** Distribution of CMV test results across different age groups Data are presented as numbers (n) and percentages (%). CMV, cytomegalovirus

Age Group	CMV Positive (n = 43)	CMV Negative (n = 119)
0-6 days	12 (17.4%)	57 (82.6%)
7-28 days	2 (18.2%)	9 (81.8%)
1 month - 18 years	3 (50%)	3 (50%)
>18 years	26 (34.2%)	50 (65.8%)
Total	43	119

Association between underlying disease and CMV positivity

Among the underlying diseases and conditions analyzed in the 43 CMV-positive patients, seven (16.3%) were renal transplant recipients, who presented with symptoms suggestive of graft rejection one year after transplantation.

IBD was the most frequently observed comorbidity in our study, seen in six patients (13.9%), followed by hematological malignancies in five patients (11.6%). Three patients had pulmonary tuberculosis (6.9%). Autoimmune diseases were present in two patients (4.7%), which included one case of systemic lupus erythematosus and one case of psoriasis. Ulcerative colitis and HIV infection were each observed in two patients (4.7% each) (Table 3). The other 16 CMV-positive patients had no underlying diseases or comorbidities. 

**Table 3:** Association of underlying diseases and conditions with CMV positivity Data are presented as numbers (n) and percentages (%). Note: The remaining 16 (37.2%) CMV-positive patients had no underlying diseases. CMV, cytomegalovirus

Underlying Diseases and Conditions	CMV Positive Patients (n)	Percentage (%)
Autoimmune diseases	2	4.7%
Pulmonary tuberculosis	3	6.9%
Ulcerative colitis	2	4.7%
Inflammatory bowel disease	6	13.9%
HIV infection	2	4.7%
Hematological malignancies	5	11.6%
Post-transplant status	7	16.3%

Association between clinical manifestations and CMV positivity

Among the CMV-positive patients, fever was the most common presenting symptom, which was observed in 19 patients (44.2%). Respiratory distress was seen in 15 patients (34.9%), while preterm birth was documented in 12 patients (27.9%). Gastrointestinal manifestations were also notably present, which included diarrhea in eight patients (18.6%), abdominal pain in seven patients (16.3%), gastrointestinal bleeding in four patients (9.3%), and vomiting in three patients (6.9%). All the patients who presented with gastrointestinal manifestations were adults (n = 22). Although these clinical features were frequently observed among CMV-positive patients, none of them showed a statistically significant association with CMV positivity (p > 0.05 for all comparisons) (Table 4).

**Table 4:** Comparison of clinical manifestations between CMV positive and CMV negative patients Data are presented as numbers (n) and percentages (%). Statistical significance was assessed using Pearson’s chi-square test with Yates’ continuity correction, except for gastrointestinal bleeding, for which Fisher’s exact test was used due to small cell counts. A p-value of < 0.05 was considered statistically significant. Note: Clinical manifestations were not mutually exclusive; individual patients may have presented with more than one symptom. CMV, cytomegalovirus

Clinical Manifestations	CMV Positive (n)	CMV Negative (n)	χ²-value	p-value
Fever	19 (44.2%)	35 (29.4%)	2.473	0.115
Abdominal pain	7 (16.3%)	25 (21.0%)	0.197	0.656
Vomiting	3 (6.9%)	20 (16.8%)	1.764	0.184
Diarrhea	8 (18.6%)	23 (19.3%)	0.000	1.000
Gastrointestinal bleeding	4 (9.3%)	6 (5.0%)	N/A	0.531
Respiratory distress	15 (34.9%)	44 (36.9%)	0.004	0.952
Preterm birth	12 (27.9%)	22 (18.5%)	1.170	0.279

Viral load distribution among CMV-positive patients

Viral load analysis done among the CMV-positive patients (n = 43) showed that the majority of them had 50 to <10,000 copies/mL of CMV DNA, which accounted for 53.5%, whereas 30.2% had >10,000 to <1,000,000 copies/mL. Around 16.3% of them had >1,000,000 copies/mL (Table 5).

**Table 5:** Viral load distribution among CMV positive patients Data are presented as numbers (n) and percentages (%). CMV, cytomegalovirus

Viral Load Category (copies/mL)	Number of Patients (n)	Percentage (%)
50 - <10,000	23	53.5%
>10,000 - <1,000,000	13	30.2%
>1,000,000	7	16.3%
Total	43	100%

Treatment and outcomes observed

All 43 positive patients were initiated on antiviral therapy according to standard guidelines, with intravenous ganciclovir (5 mg/kg in two divided doses for two to three weeks), followed by oral valganciclovir if viremia persisted. Follow-up testing with CMV quantification showed below detectable levels (<50 copies/mL) in 13 (30.2%) patients, while repeat qPCR was not done in the remaining 30 (69.8%) patients, as they improved clinically, indicating treatment efficacy.

## Discussion

This study identified an overall CMV prevalence of 26.5% (43/162) among clinically suspected patients in a tertiary care center. This finding is consistent with other Indian studies, such as the recent report by Fomda et al. (2025) [14].

Although CMV positivity was higher among pediatric patients (50%) and adults (34.2%), neonates accounted for a considerable proportion (17.5%) of CMV-positive cases in the study population. This finding highlights the importance of CMV surveillance in neonates. Clinically, these neonates were small for gestational age and presented with sepsis-like illness. This supports the systematic review by Salomè et al. (2023), which reported that many congenital CMV infections are asymptomatic, while symptomatic cases are strongly linked to intrauterine growth restriction (IUGR) and neurodevelopmental delays [15]. Another study on low-birth-weight newborns in an Indian tertiary care setting found a significant association between CMV positivity and IUGR [16]. This reinforces our study observation that early PCR screening may be useful in neonates with unexplained growth failure, as timely detection could help reduce long-term sequelae, like sensorineural hearing loss.

Among the positive cases, 51.1% (n = 22) of patients presented with gastrointestinal symptoms, including diarrhea, abdominal pain, vomiting, and gastrointestinal bleeding. A few of them had underlying ulcerative colitis or IBD. This is a significant observation, as CMV colitis is a well-documented complication that can mimic or exacerbate IBD. Recent reviews from 2024 highlight the diagnostic difficulty in immunocompetent and immunosuppressed adults, where CMV often presents as refractory gastrointestinal bleeding or colitis [17]. Our data support the necessity of screening for CMV in patients presenting with refractory gastrointestinal symptoms, as steroid treatment for presumed IBD can exacerbate an underlying CMV infection [18].

In our study, renal transplant recipients and patients with hematological malignancies constituted 16.3% and 11.6% of the positive cases, respectively. CMV remains a major concern for graft survival. Roy et al. (2024) reported similar findings in renal transplant recipients, stating that CMV remains a major cause of graft rejection and nephritis [19].

The use of quantitative PCR in our setting helped in early detection and initiation of pre-emptive therapy with ganciclovir, which successfully reduced viral loads. This is consistent with a recent study by Lin et al. (2023), which reported that ganciclovir helped in reducing inflammatory reactions and improving immune functions [20]. In addition, another study by Reed et al. (2023) emphasized that prophylactic ganciclovir, given before transplant, significantly reduces CMV infection in the post-transplant period [21].

Limitations

This retrospective, single-center study included only clinically suspected and high-risk patients. This may have introduced selection bias and reduced generalizability, as cases with asymptomatic CMV reactivation could have been missed. The inclusion of heterogeneous patient groups, such as neonates, transplant recipients, and immunocompromised adults, may have influenced prevalence estimates. The relatively smaller sample size limited detailed analysis across individual risk categories. A multicenter study, involving more patients and an extended duration of study, could have provided more definitive statistical results. Even though real-time PCR was useful in detecting active infection, uniform viral load thresholds and tissue confirmation were not consistently available. Long-term follow-up was beyond the scope of this study.

## Conclusions

This retrospective analysis suggests that CMV is an important contributor to morbidity in a tertiary care setting, especially among neonates and immunocompromised individuals. It also indicates a notable burden of active infection in clinically suspected cases. CMV positivity was strongly associated with immunosuppression and renal transplantation, highlighting CMV as an important opportunistic pathogen in these high-risk groups. Gastrointestinal manifestations emerged as a prominent clinical presentation in this study. This highlights the need to consider CMV as a differential diagnosis in patients receiving immunosuppressive therapy who present with refractory gastrointestinal symptoms, such as abdominal pain, vomiting, and diarrhea, that do not respond to routine treatment.

The study also reinforces the importance of real-time PCR in the early detection and management of CMV infection in tertiary care hospitals. It also suggests that viral load-based screening, particularly in neonates and immunocompromised patients, may be helpful in reducing CMV-associated complications and improving clinical outcomes.
